# The Relation Between Vocal Pitch and Vocal Emotion Recognition Abilities in People with Autism Spectrum Disorder and Typical Development

**DOI:** 10.1007/s10803-018-3681-z

**Published:** 2018-07-18

**Authors:** Stefanie Schelinski, Katharina von Kriegstein

**Affiliations:** 1 Max Planck Institute for Human Cognitive and Brain Sciences, Stephanstraße 1a, 04103 Leipzig, Germany; 2 Technische Universität Dresden, Faculty of Psychology, Bamberger Straße 7, 01187 Dresden, Germany

**Keywords:** Autism spectrum disorder, Voice, Emotion recognition, Pitch, Auditory, AQ

## Abstract

We tested the relation between vocal emotion and vocal pitch perception abilities in adults with high-functioning autism spectrum disorder (ASD) and pairwise matched adults with typical development. The ASD group had impaired vocal but typical non-vocal pitch and vocal timbre perception abilities. The ASD group showed less accurate vocal emotion perception than the comparison group and vocal emotion perception abilities were correlated with traits and symptoms associated with ASD. Vocal pitch and vocal emotion perception abilities were significantly correlated in the comparison group only. Our results suggest that vocal emotion recognition difficulties in ASD might not only be based on difficulties with complex social tasks, but also on difficulties with processing of basic sensory features, such as vocal pitch.

**Electronic supplementary material:**

The online version of this article (10.1007/s10803-018-3681-z) contains supplementary material, which is available to authorized users.

## Introduction

Successful interaction with other people can critically rely on vocal information. The voice conveys the speech message, provides information about who a person is (voice identity) and also about the speaker’s emotional state (for review see Belin et al. 2004). Expressing emotions by voice is an evolutionary preserved process (Darwin 2009; Talkington et al. 2013; Vettin and Todt 2005) and the correct interpretation of emotional calls from conspecifics can be critical for survival (Manser 2001; Ordonez-Gomez et al. 2015; Seyfarth et al. 1980). The perception of vocal emotion (i.e. the emotional information conveyed in a speaker’s voice) in humans relies on the analysis of specific acoustic features of the voice, such as the fundamental frequency (F0; i.e. the lowest frequency within the speech signal) or sound intensity (Fairbanks and Pronovost 1938; Gold et al. 2012; Quam and Swingley 2012). The fundamental frequency is perceived as vocal pitch (i.e. the perceptual correlate of F0) and sound intensity is perceived as loudness respectively.

There is evidence that people with autism spectrum disorder (ASD) have difficulties in recognising emotions and mental states from vocal speech (Globerson et al. 2015; Golan et al. 2007; Philip et al. 2010; Rosenblau et al. 2017; Rutherford et al. 2002; for review see Lartseva et al. 2015; but see Jones et al. 2011; Xavier et al. 2015).

It is currently unclear why people with ASD have difficulties with recognising vocal emotion. On one hand the difficulties might be based on a perceptual processing deficit, i.e. a deficit in perceiving voice acoustic features, such as impaired pitch perception. Alternatively, the difficulties might be due to higher-level social cognition difficulties. This latter view was supported by a recent study (Globerson et al. 2015), which is to our knowledge the only previous study that investigated the relation between abilities for perception of acoustic features and for vocal emotion recognition in people with ASD. The authors found that vocal emotion recognition was impaired in adults with high-functioning ASD, but that pitch discrimination (i.e. the ability to detect differences in pitch) for sounds was intact and positively correlated with vocal emotion recognition abilities. The authors concluded that the vocal emotion recognition deficit in people with ASD is associated with higher-level cross-modal emotion difficulties and difficulties of social cognition and that auditory perceptual abilities help to compensate for these higher-level emotion recognition difficulties (Globerson et al. 2015). However, in that study pitch discrimination was tested with non-vocal sounds (i.e. pure tone sine wave tones) and there is recent evidence that adults with high-functioning ASD have deficits in pitch discrimination rather for vocal (i.e. speech including vowels and words), than for non-vocal sounds (Jiang et al. 2015; Schelinski et al. 2017). This finding reopens the possibility that the difficulties with vocal emotion recognition in people with ASD are based on perceptual difficulties. We here hypothesised a relation between vocal emotion processing and pitch discrimination in *vocal* sounds. Such a finding would be in line with the view that altered sensory processing in people with ASD might be critically contributing in explaining non-social (e.g. Pellicano 2013) as well as social symptoms associated with ASD (e.g. Baum et al. 2015; Robertson and Baron-Cohen 2017). Although sensory dysfunctions are now also integral parts in the DSM-5, sensory contributions to ASD symptomatology and impairments in higher social cognition have been poorly characterised and are often focused on hyper- and hypo-sensory processing which usually refers to an enhanced ability to perceive sensory stimuli or absent or less response to sensory input (for reviews see e.g. Pellicano 2013; Robertson and Baron-Cohen 2017).

To test our hypothesis, we investigated vocal emotion recognition and vocal and non-vocal pitch perception in a group of adults with high-functioning ASD and typically developing matched comparison group participants. We additionally included a test on vocal timbre discrimination to investigate whether the relation to vocal emotion processing would be related more specifically to the perception of vocal pitch or more general to the perception of voice acoustic features, such as vocal timbre (i.e. the property that distinguishes two sounds of identical pitch, intensity, duration and location; see e.g. Griffiths and Warren 2004).

Difficulties in emotion recognition are associated with reduced social functioning (e.g. Couture et al. 2006; Garcia-Villamisar et al. 2010). For example, difficulties in emotion recognition have been associated with lower social adaptive behaviour in people with ASD (Garcia-Villamisar et al. 2010). Investigating vocal emotion recognition in people with ASD is important because it will enhance the understanding of the underlying mechanisms of difficulties in socially relevant auditory processing. This better understanding might contribute to the identification of diagnostically relevant features as well as to more informed counselling and therapy strategies for emotion recognition difficulties in people with ASD.

## Methods

### Participants

We tested 16 adults with ASD (ASD group) and 16 typically developing adults (comparison group). The groups were matched pairwise, i.e. each comparison group participant was matched to one participant in the ASD group with respect to gender (male or female), chronological age (age difference within each participant pair ≤ 3 years), handedness (right or left as assesed by a standard questionnaire; Oldfield 1971), and intelligence quotient [IQ; Table 1; Full-scale IQ difference within each participant pair was maximally one standard deviation (15 IQ points)]. IQ was assessed using the German adapted version of the Wechsler Adult Intelligence Scale (WAIS-III; Wechsler 1997; German version by von Aster et al. 2006). We used the WAIS-III, because the adapted German version of the latest version (WAIS-IV; Petermann 2012) became available when the participant testing for the present study was already on-going. All participants had an IQ within the normal range or above (IQ > 85), indicating that all participants were on a ‘high-functioning’ cognitive level. Additionally, groups showed comparable concentration performances (d2 test of attention; Brickenkamp 2002; Table 1).

**Table 1:** Descriptive data for the ASD (*n* = 16) and the comparison group (*n* = 16) and group comparisons

Characteristic	ASD group		Comparison group		
Gender	13 male, 3 female		13 male, 3 female		
Handedness^a^	14 right, 2 left		14 right, 2 left		

	*M*	*SD*	*M*	*SD*	*p*
Age	33.75	10.12	33.69	9.58	0.986
Range	20–51	20–51	18–52	18–52	
WAIS-III^b^ scales					
Full-scale IQ	110.31	13.79	111.50	10.97	0.789
Verbal IQ	110.75	12.35	108.75	12.59	0.653
Performance IQ	107.38	17.55	112.69	9.59	0.296
Working memory	108.63	2.22	108.00	3.76	0.887
d2 test of attention^c^	104.19	8.61	106.06	3.41	0.645
AQ^d^	39.81	6.61	14.13	4.77	< 0.001^*^
Range	26–48	26–48	5–23	5–23	

Each participant in the comparison group was matched with respect to chronological age, gender, intelligence quotient (IQ), and handedness to the profile of one ASD group participant (*M* = mean; *SD* = standard deviation)

*Significant group difference (*p* < .05)

^a^Handedness was assessed using the Edinburgh handedness questionnaire (Oldfield 1971)

^b^*WAIS-III* Wechsler Adult Intelligence Scale, 3rd version (Wechsler 1997; German adapted version: von Aster et al. 2006; *M* = 100; *SD* = 15)

^c^d2 Test of Attention (Brickenkamp 2002; *M* = 100; *SD* = 10)

^d^*AQ* Autism Spectrum Quotient (Baron-Cohen et al. 2001; German version adapted from Freitag et al. 2007; http://kriegstein.cbs.mpg.de/AQ/AQ_Deutsch_Schelinski.pdf). A total score of 32+ is considered a useful cut-off for distinguishing individuals who have clinically relevant levels of traits associated with the autism spectrum (Baron-Cohen et al. 2001)

All participants filled out the autism spectrum quotient (AQ; Baron-Cohen et al. 2001; German version adapted from Freitag 2010; Freitag et al. 2007; Table 1).

All participants reported normal hearing abilities and no limitations or disorders associated with the ear or hearing. Normal hearing abilities were confirmed with pure tone audiometry (hearing level equal or above 25 dB at the frequencies of 250, 500, 1000, 1500, 2000, 3000, 4000, 6000, and 8000 Hz tested in each ear separately). All participants were native German speakers. All were free of medication except two participants taking a histamine antagonist for allergies (1 control, 1 ASD) and two participants taking antihypertensive medication (2 ASD). None of the participants reported to have a neurological disease. Two participants in the ASD group reported a history of a depressive episode and another participant in the ASD group reported a comorbid diagnosis of social phobia. There were no further comorbidities reported by the participants or stated in the medical reports. Three additional participants in the ASD group were not included in the analysis due to incidental findings in an anatomical MRI-scan (for details see Schelinski et al. 2016). We also excluded the comparison group participants that were matched to these participants’ profiles in the ASD group.

We recruited people with ASD via autism outpatient clinics and announcements in communities for people with ASD, i.e. self-help groups and online fora. Participants in the ASD group had previously received a formal clinical diagnosis of Asperger syndrome (11 male, 3 female) or childhood autism (2 male, Verbal-IQ 100 and 119) according to the diagnostic criteria of the International and Statistical Classification of Diseases and Related Health Problems (ICD-10; World Health Organisation 2004). We only included participants into the ASD group who could provide a clinical diagnosis. That means that the diagnoses of all ASD participants were made by independent clinical experts before participating in the study. Additionally, the diagnoses for all participants in the ASD group were corroborated with the Autism Diagnostic Observation Schedule (ADOS; Lord et al. 2000; German version by Rühl et al. 2004) and, if caregivers were available (*n* = 9), additionally with the Autism Diagnostic Interview-Revised (ADI-R; Lord et al. 1994; German version by Bölte et al. 2003) and the Social Communication Questionnaire (SCQ; Rutter et al. 2003; German version by Bölte and Poustka 2006; Table 2). ADOS and ADI-R measures reported in the current study were performed by the first author, who is a psychologist with formal training on administration of these tests.

**Table 2:** Overview of diagnostic scores in the ASD group

Diagnostic test	*M* cut-offs for autism/autism spectrum	*SD*
Participants as informant		
Interview [ADOS^a^ (*n* = 15)]		
Social interaction and communication	11.00 (12/7)	2.78
Social interaction	7.20 (7/4)	1.97
Communication	3.80 (3/2)	1.27
Parents as informant		
Questionnaire [SCQ^b^ (*n* = 9)]	20.33 (15)	5.70
Interview [ADI-R^c^ (*n* = 9)]		
Social interaction and communication	36.22	8.04
Social interaction	21.11 (17)	5.09
Communication	13.89 (8)	4.37

*M* mean, *SD* standard deviation

^a^*ADOS* Autism Diagnostic Observation Schedule (Lord et al. 2000; German version: Rühl et al. 2004)

^b^*SCQ* Social Communication Questionnaire (Rutter et al. 2003; German version: Bölte and Poustka 2006)

^c^*ADI-R* Autism Diagnostic Interview-Revised (Lord et al. 1994; German version: Bölte et al. 2003)

We recruited the comparison group participants from the participant database of the Max-Planck-Institute for Human Cognitive and Brain Sciences Leipzig. The database contains participants who have contacted the institute because they are interested in taking part in scientific studies. The database contains volunteers with e.g. different age ranges and different socioeconomic status or educational backgrounds. Participants in the comparison group reported to have no neurological or psychiatric history and no family history of ASD. None of the comparison group participants exhibited a clinically relevant number of traits associated with ASD as assessed by the AQ (Baron-Cohen et al. 2001; German version adapted from Freitag 2010; Freitag et al. 2007; Table 1). All participants were told that they take part in a study on voice perception which includes several computer based tasks on voice, speech and sound perception. All participants gave written informed consent in accordance with procedures approved by the Research Ethics Committee of the University of Leipzig.

### Experiment

The experiment included tests on vocal emotion recognition, vocal pitch and vocal timbre discrimination and the perception of non-vocal pitch. The vocal emotion recognition, vocal pitch and vocal timbre discrimination tests were carried out under the same conditions in a quiet room. During these tests the experimenter was present in the room, but separated from the participant by a partition panel. Auditory stimuli were presented using Sennheiser HD 201 head phones (Sennheiser, Wedemark, Germany) at 65 dB sound pressure level (sound level meter SL-4001, Lutron Electronic, China). For all experiments, participants were seated in a comfortable chair facing a computer screen placed at approximately 30 cm distance. Participants completed the non-vocal pitch perception test (online-test) at home. The tests were part of a larger study that also included tests on voice identity recognition, voice discrimination, speech recognition, musical instrument, and face recognition. The results of voice identity recognition and their relation to the results in vocal pitch, vocal timbre and non-vocal pitch perception have been reported previously (Schelinski et al. 2017).

### Vocal Emotion Recognition Test

#### Overview

To test the ability to recognise emotions from voice, participants decided whether auditorily presented words were spoken in a neutral manner or in a way expressing the emotions happiness, sadness, fear, anger, or disgust (Ekman 1972; Oatley and Johnson-Laird 1987).

#### Stimuli

The stimuli included 134 two-syllable semantically neutral German nouns [e.g. ‘Reihe’ (English: ‘row’), ‘Bericht’ (English: ‘report’), or ‘Dreieck’ (English: ‘triangle’). Words were spoken by one female and one male professional actor in Standard German (44,100 Hz sampling rate, resolution of 16 bits). Words were spoken in a way expressing the following emotions: happiness, sadness, fear, anger, disgust, or in a neutral way (henceforth we refer to all six expression including the neutral words as emotion). The expressed emotion was independent from the semantic meaning of the words. Stimuli were taken from a validated database developed for vocal emotion perception studies (Wendt 2007; Wendt and Scheich 2002). Words were included in the final corpus of this database if the level of acceptance, i.e. the assignment of an emotion to every word (*n* = 74 raters, 18–62 years old) was higher than 70% (Wendt 2007). Different words were presented for each emotion and all words were validated for semantic neutrality (Wendt 2007). We adjusted the stimuli to the same root mean square (rms = 0.05) using Matlab (version 7.7, The MathWorks, Inc., USA).

#### Experimental Design

In each trial, a word spoken in one of the six vocal emotions was presented via headphones. After the auditory stimulus, all six emotions were presented as written words on the screen and participants were instructed to decide in which emotion the word was expressed. Written words were presented until participants gave a response. Each emotion was presented 20 times, 10 times spoken by the female and 10 times spoken by the male speaker (120 trials in total). All words were presented in randomised order. In order to familiarise the participants with the test and speakers, examples from each emotion spoken by the two speakers and two example trials were presented before the test. Completing the test took approximately 20 min.

### Vocal Pitch and Vocal Timbre Discrimination Test

#### Overview

To test the ability to recognise changes in vocal pitch, participants performed a vocal pitch discrimination test. To test the ability to recognise changes in voice timbre, participants performed a vocal timbre discrimination test. In both the vocal pitch and the vocal timbre discrimination test, the source speech material was the same, only the manipulation of the speech signal differed.

Vocal pitch is a correlate of the vibration rate of the vocal folds of a speaker (Hanson and Chuang 1999; Smith and Patterson 2005; for an overview see Kreiman and Sidtis 2011). Thus, in the vocal pitch discrimination test, the frequency of the glottal pulse rates of the speech signal (F0) were manipulated in order to simulate different pitches while keeping the vocal tract length (VTL) constant. F0 of a voice is the primary determinant of the perceived pitch. The mean F0 determines whether we perceive a voice as rather high or low. For example, the mean F0 for men is 115 and 220 Hz for women (see Kreiman and Sidtis 2011). That means that for an average female speaker, the vocal folds open and close 220 times per second and less for an average male speaker, i.e. 115 times/s. As a consequence, we usually perceive an average female voice as higher in pitch than a male voice. Listeners can usually perceive very small changes of the mean F0 of as little as 2% (2.4 Hz, e.g. Smith et al. 2005).

A major aspect of vocal timbre is determined by the VTL of a speaker. Differences in VTL are perceived as differences in speaker height (Fitch and Giedd 1999; Smith et al. 2005). For example, adult men usually have longer VTLs than children or women (Fitch and Giedd 1999). In the vocal timbre discrimination test, the frequencies of the prominent spectral peaks (formants) were shifted in order to simulate different speaker sizes that are perceived as differences in voice timbre (Smith and Patterson 2005; Smith et al. 2005).

In both the vocal pitch and the vocal timbre discrimination tests, we used an adaptive tracking procedure, i.e. the task difficulty in a respective trial was adapted to the response in the previous trial. In both tests we measured the individual thresholds where participants were still able to differentiate between two vocal speech stimuli based on the difference in perceived vocal pitch or the perceived vocal timbre. We measured ΔF0 which is a change in F0 from the first to second stimulus within a trial in the vocal pitch discrimination test and changes in the spectral envelope ratio (ΔSER) in the vocal timbre discrimination test (for more details see experimental design).

#### Stimuli

In both tests, stimuli consisted of five vowels (/a/, /e/, /i/, /o/, /u/) spoken by one male speaker (44,100 Hz sampling rate, for a detailed description see Smith and Patterson 2005; Smith et al. 2005). Each stimulus was 600 ms long and manipulated using Straight software (Kawahara and Irino 2005) in a Matlab environment (version 7.7, The MathWorks, Inc., MA, USA). Note that the source speech material was the same in both tests, only the manipulation of the speech signal differed. Stimulus manipulation for the vocal pitch discrimination test: The stimuli were resynthesised in their glottal pulse rate (GPR; i.e. the average oscillation rate of the glottal folds) by shifting the fundamental frequency (F0), which is the acoustic correlate of a mean speaker’s GPR (Koyama et al. 1971; Smith et al. 2005; for an overview see Kreiman and Sidtis 2011). For each vowel, GPR was manipulated by an amount defined in musical cents (ΔF0; 100 cents = 1 semitone). The stimulus set contained manipulations of the baseline value of 112 Hz (which is near to the average for man) or 1200 cents in 1 cent step-size ranging from 0 to 2400 cents. Thus, the total stimulus set for the vocal pitch discrimination test included 12,000 sounds. Stimulus manipulation for the vocal timbre discrimination test: The stimuli were resynthesised in their acoustic effects of the VTL by changing the spectral envelope ratio (SER; Smith and Patterson 2005). For each vowel, spectral envelopes were scaled proportionally up and down in log-frequency space from the original spectral envelope. The stimulus set contained manipulations of the baseline ΔSER (12%) in 0.001 step-sizes ranging from 0.80 to 1.30. Thus, the total stimulus set for the vocal timbre discrimination test included 12,000 sounds.

#### Experimental Design

Each test contained five runs (one for each vowel). To identify individual discrimination thresholds in pitch (vocal pitch discrimination test) and timbre recognition (vocal timbre discrimination test), we measured the individual just noticeable differences (JND) using an adaptive tracking procedure (one up, one down staircase method; Kaernbach 1991) in two separate sessions. In each trial we presented two stimuli successively and participants were instructed to decide which of the two stimuli had the higher pitch (vocal pitch discrimination test) or which of the two stimuli sounded as if it was spoken by the smaller person, i.e. had the smaller body height (vocal timbre discrimination test). In each trial, the task difficulty was adapted to the preceding response: In the vocal pitch discrimination test, participants listened to two sequentially presented stimuli that only differed in their F0. One of the two stimuli always had a fixed F0 of 112 Hz (which is near to the average for man, e.g. Krook 1988; Peterson and Barney 1952) and the other differed in F0 by an amount (ΔF0) defined in musical cents (1 semitone = 100 cents). In each trial, the fixed and manipulated vowels were presented in randomised order. The initial ΔF0 was 100 cents. This value increased in steps of 30 cents following each incorrect response and decreased in steps of 10 cents following each correct response. After four reversals (a switch from a correct to an incorrect response or vice versa within two consecutive trials), the up and down step sizes were changed to 6 and 2 cents respectively and the block of trials continued for further 10 reversals. To derive the individual JNDs in cent in each run, the JND was estimated from all ΔF0 of these final 10 reversals and averaged over all five runs.

The vocal timbre discrimination test was identical to the vocal pitch discrimination test, only the stimuli manipulation and the task instructions were different. In the vocal timbre discrimination test participants listened to two sequentially presented stimuli that only differed in their SER. One of the two stimuli always had a fixed spectral envelope (equal to the spectral envelope of the original speaker), and the other differed by ΔSER, defined in percent. In each trial, the fixed and manipulated vowels were presented in randomised order. The initial trial was set to a ΔSER of 12%. This value increased in steps of 3% following each incorrect response and decreased in steps of 1% after each correct response. After four reversals, the up and down step sizes were changed to 0.6 and 0.2% respectively and the block of trials continued for a further 10 reversals. To derive the individual JNDs in each run, the JND was estimated from all ΔSER values of these final 10 reversals and averaged over all five runs.

In both tests, participants indicated whether the first or the second vowel of the vowel pair was higher (pitch test) or spoken by the smaller person (timbre test) by pressing a button (‘1’ or ‘2’) on the keyboard. During the experiment the written numbers ‘1’ or ‘2’ were presented on the screen. After each response immediate feedback was provided. Here, the chosen number changed from white to green font if the response was correct or to red font if the response was incorrect. Before the test sessions, participants were presented with two stimuli representing extremes of the F0 range (vocal pitch discrimination test) and two extremes of the SER range (vocal timbre discrimination test) range in order to familiarise participants with the stimulus manipulation. The total duration for each test was approximately 15 min. Both tests were presented consecutively whereby the order of tests was randomised across the subjects in each group, but the same for the matched TD-ASD group pairs. Both tests were implemented in Python (version 2.7.3, http://python.org/).

### Non-vocal Pitch Perception Test

We used an online-version of the Montreal Battery of Evaluation of Amusia (MBEA; Peretz et al. 2003, 2008). This online-version includes two tests on musical pitch perception: ‘scale’ and ‘out-of-key’.

#### Stimuli

The online-version we used in our study is available at http://www.brams.umontreal.ca/onlinetest/. The test contains different melodies presented with ten different timbres (e.g. piano, guitar, or harp) taken from the MBEA (Peretz et al. 2003).

#### Experimental Design

In the ‘scale’ and the ‘out-of-key’ subtests, judging differences in pitch are critical: In the ‘scale’ subtest pairs of melodies are presented. Some of them are scale-violated versions of the comparison melody, i.e. the pitch is out of scale while retaining the original melodic contour. Participants have to decide whether the two melodies are the same or different. This test included 30 trials. In the ‘out-of-key’ subtest single melodies are presented, some of them containing one tone that is mistuned. Participants have to decide whether the melody contains a tone that is out of the key with the rest of the melody. This test included 24 trials. Participants completed the online-test at home. We provided German instructions of all information given in the original test. Completing the online-test including an additional test on meter recognition (see Schelinski et al. 2017) took approximately 15 min.

### Statistical Analyses

We analysed the data using SPSS version 24 (IBM SPSS Statistics, NY, USA). Statistical tests were calculated two-tailed if not otherwise stated (i.e. one-tailed if we had an a priori directed hypothesis). The level of significance was defined at *α* = 0.05. If not otherwise stated, all analyses included data from 16 participants with ASD and their matched comparison group participants. Data from three participants with ASD were not available for the non-vocal pitch perception test.

#### Group Differences

To test group differences in the vocal emotion recognition test, we computed a 2 × 6 factorial ANOVA with the between-subject factor ‘group’ (comparison group, ASD group) and the within-subject factor ‘emotion’ (happiness, sadness, fear, anger, disgust, neutral). For post-hoc testing and all other group comparisons we computed independent *t*-tests. In the vocal emotion recognition test, we used the percentage of correct responses as dependent variable. For the vocal pitch and timbre discrimination tests, we used the threshold values for the just noticeable differences in cent (in the vocal pitch discrimination test) or in SER (in the vocal timbre discrimination test) as dependent variables. For the non-vocal pitch perception test we used averaged scores over the ‘scale’ and the 'out-of-key’ test as dependent variables.

#### Correlation Analyses

We used Pearson’s correlation coefficient for correlation analyses. We used Spearman’s rho for not normally distributed variables (Shapiro–Wilk test). For correlation analyses we did not include outliers. We identified outliers for each test and group separately. We defined outliers as cases who scored outside 1.5 times the interquartile range (Tukey 1977), implemented as a standard procedure in SPSS version 24 (PASW Statistics, IBM SPSS Statistics, NY, USA). One participant with ASD was an outlier because of low performance in the vocal pitch discrimination test and the non-vocal pitch perception test and this participant was therefore excluded from the correlation analyses for these tests. There were no further outliers in the ASD or the comparison group. For correlation analyses including the vocal emotion recognition test we used the total score pooled over all six emotions.

#### Hypotheses

Based on previous findings (Globerson et al. 2015; Golan et al. 2007; Philip et al. 2010; Rosenblau et al. 2017; Rutherford et al. 2002) we expected the ASD group to perform worse compared to the comparison group on vocal emotion recognition. Based on previous research on vocal emotion recognition and its relation to vocal pitch (e.g. Fairbanks and Pronovost 1938; Gold et al. 2012; Quam and Swingley 2012; Scherer et al. 1991) we expected that in people with typical development, performance in vocal emotion recognition and pitch discrimination would be correlated positively, i.e. better performance in vocal emotion recognition would be associated with better performance in vocal pitch discrimination. For the ASD group we considered two possible outcomes: A correlation between vocal emotion recognition and pitch discrimination abilities as assumed for people with typical development might indicate similar mechanisms in both groups, whereas no correlation between the two measures might indicate at least partially different mechanisms. We did not expect correlations between vocal emotion recognition and control tests (i.e. vocal timbre and non-vocal pitch discrimination test).

### Behavioural and Acoustic Characterisation of the Stimuli

The perception of vocal emotion is determined by acoustic voice features, such as the frequency range (Fairbanks and Pronovost 1938). We therefore determined the frequency range of the stimuli (Supplementary Methods). A previous study showed that performance in vocal emotion recognition in people with ASD is associated with the emotional intensity of the stimulus, i.e. the probability that a certain speech stimulus is recognised as a certain emotion (Globerson et al. 2015). In the study by Globerson et al. (2015), the emotional intensity of the stimulus was assessed by the recognition accuracy in a vocal emotion recognition test in an independent sample of 20 participants. Similarly, we additionally determined the level of emotional intensity of the stimuli in an independent sample of 21 adult participants with typical development (Supplementary Methods). To test whether the recognition accuracy in the vocal emotion recognition test was influenced by the level of emotional intensity or the frequency range of the stimulus material, we additionally conducted two separate ANOVAs for emotional intensity and frequency range including the within group factor ‘emotional intensity level’/‘frequency range’ (very low, low, high, very high) and the between group factor ‘group’ (comparison group, ASD group).

## Results

### Vocal Emotion Recognition is Impaired in the ASD Group

**Fig. 1:**
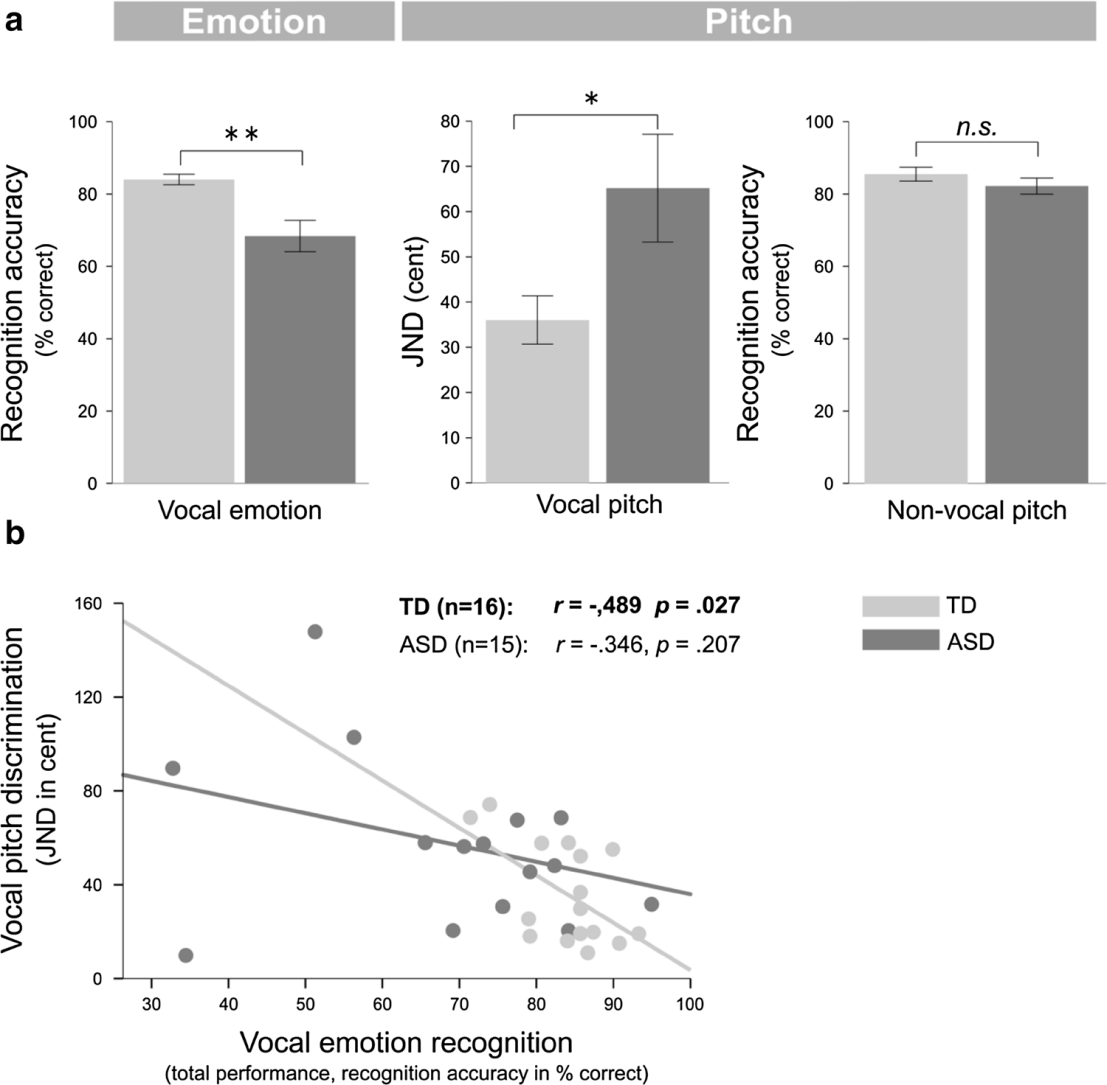
Results of the vocal emotion recognition test and tests on pitch perception. **a** The ASD group performed worse than the comparison group with typical development (TD) in recognising vocal emotion and discriminating vocal pitch. There were no significant group differences for the non-vocal pitch perception test. **b** In the comparison group, performance in the vocal pitch discrimination test correlated negatively with performance accuracy in the vocal emotion recognition test, implicating that better vocal pitch discrimination abilities were associated with better vocal emotion recognition abilities. There was no such significant correlation in the ASD group. *JND* just noticeable difference. Note that smaller JNDs indicate better performance. Error bars represent ± 1 SE; *p < .05; *n.s*. not significant

In the vocal emotion recognition test, the average total accuracy over all six emotions (total performance) was 68.33% in the ASD group (*n* = 16) and 83.95% in the comparison group (*n* = 16) (Fig. 1a). An ANOVA for the factors group and emotion revealed significant main effects for the factors group (*F*(1,30) = 11.594, *p* = .002; *η*^2^_*p*_ = .279) and emotion (*F*(5,26) = 15.062, *p* < .001; *η*^2^_*p*_ = .743). There was no significant interaction between the factors group and emotion (*F*(5,26) = 2.346, *p* = .069; *η*^2^_*p*_ = .311). We additionally explored whether there were group differences for the single emotions (Table 3): Post-hoc testing showed that there was no significant group difference when the word was expressed in a neutral manner (*t*(30) = 0.943, *p* = .353; *d* = 0.333). The ASD group performed significantly worse than the comparison group for the emotions sadness (*t*(30) = 3.573, *p* = .001; *d* = 1.263) and fear (*t*(30) = 3.002, *p* = .005; *d* = 1.061) (Bonferroni corrected for the six emotions, Table 3). There were also trends towards worse performance for the ASD as compared to the comparison group for the emotions happiness, anger, and disgust (all *p*s < .054; Table 3). For the interested reader we provide an overview of the percentage of correct and incorrect (confusion) choices for each emotion for both groups in the Supplementary Material (Supplementary Fig. 1; Supplementary Table 1).

**Table 3:** Overview of the average recognition accuracy scores for the different emotions and the total score in the vocal emotion recognition test

	ASD group (*n* = 16)		Comparison group (*n* = 16)		*p*
	*M*	*SD*	*M*	*SD*	
Happiness	64.10	30.93	80.54	9.93	0.052
Sadness	51.45	32.05	81.64	10.76	0.001*
Fear	68.37	27.82	89.85	6.71	0.005*
Anger	84.98	13.15	92.48	6.58	0.050
Disgust	50.18	22.77	65.97	21.78	0.054
Neutral	90.63	9.11	93.13	5.43	0.353
Total score	68.33	17.41	83.95	5.86	0.002*

*Significant group differences Bonferroni corrected for the six emotions at *p* < .008

### Significant Correlation Between Vocal Emotion and Vocal Pitch Perception Within the Comparison Group

The ASD group showed impaired vocal pitch discrimination abilities as compared to the comparison group, but there were no significant group differences in tests on vocal timbre discrimination and non-vocal pitch perception (Schelinski et al. 2017; Fig. 1a; Table 4). Correlation analyses between the total performance in the vocal emotion recognition test and the JND in the vocal pitch discrimination test revealed a significant negative correlation in the comparison group (*r* = − .489, *p* = .027, *n* = 16; one-tailed; Spearman correlation; Fig. 1b). This negative correlation indicated that in the comparison group, better vocal pitch discrimination abilities were associated with better performances in vocal emotion recognition. In the ASD group, there was no significant correlation between the vocal emotion and the vocal pitch discrimination test (*r* = − .346, *p* = .207, *n* = 15). There was no significant difference between the correlation coefficient (i.e. correlation between vocal emotion and vocal pitch) of the comparison group and the same correlation coefficient (i.e. correlation between vocal emotion and vocal pitch) of the ASD group (Fisher’s *Z*-test: *Z* = − 0.66, *p* > .05, for *r* = − .554 using Pearson’s correlation within the comparison group).

**Table 4:** Summary of average scores for tests on vocal timbre discrimination and non-vocal pitch perception reported in Schelinski et al. (2017)

	ASD group		Comparison group		*p*
	*M*	*SD*	*M*	*SD*	
Test					
Vocal pitch discrimination (JND^a^ in cent)	65.18	47.69	36.02	21.39	0.033*
Vocal timbre discrimination (JND^a^ in SER)	4.28	2.17	3.45	1.62	0.231
Non-vocal pitch perception (MBEA^b^; % correct)	82.27	8.03	85.56	7.57	0.267

Scores are summarised as average over group with standard deviation (*SD*) and *p*-values from independent *t*-tests

*Significant group differences (*p* < .05)

^a^*JND* just noticeable difference. Note that smaller JNDs indicate better performance

^b^*MBEA* online-version of the Montreal Battery of Evaluation of Amusia (Peretz et al. 1994, 2003, 2008)

Correlation analyses between the vocal emotion recognition and control tests revealed that there were no significant correlations of the vocal emotion recognition performance with the vocal timbre discrimination test (*n* = 16), nor the non-vocal pitch discrimination test (*n* = 15) (all *p*s > .26). Also the ASD group did not show these correlations (*n* = 13 for correlation with non-vocal pitch performance, *n* = 16 for correlation with vocal timbre performance; all *p*s > .3). There was a trend to significance for the difference between the correlation coefficient for vocal emotion and vocal pitch (i.e. correlation between vocal emotion and vocal pitch) and the correlation coefficient between vocal emotion and non-vocal pitch (i.e. correlation between vocal emotion and non-vocal pitch) within the comparison group (Steiger’s *Z* test: *Z* = 0.55, *p* > .05). There were no further significant differences between the correlation coefficient for vocal emotion recognition and the correlation coefficient for vocal pitch discrimination (i.e. correlation between vocal emotion and vocal pitch) and the correlation coefficient for vocal emotion recognition with any of the other two acoustic features (i.e. correlation between vocal emotion and non-vocal pitch within the ASD group; correlation between vocal emotion and vocal timbre within the ASD group; correlation between vocal emotion and vocal timbre within the comparison group) (Steiger’s *Z* tests, all *p*s for both groups > .178).

### Correlation Between Vocal Emotion Recognition and the Extent of Traits Associated with the Autism Spectrum

We used the AQ as a self-administered questionnaire to assess the extent of traits associated with the autism spectrum in participants with typical development and participants with ASD (Baron-Cohen et al. 2001; Freitag et al. 2007). Additional exploratory analyses revealed that for the comparison group, there was a significant correlation between the total performance in the emotion recognition test and the AQ score (*r* = − .501, *p* = .048, *n* = 16). This correlation indicates that lower self-reported traits associated with the autism spectrum were associated with higher scores in the vocal emotion recognition test in people with typical development. There was no such correlation for the ASD group (AQ score: *r* = − .197, *p* = .465, *n* = 16). There were no correlations with the vocal pitch discrimination test for the AQ score (comparison group: *r* = .377, *p* = .149, *n* = 16; ASD group: *r* = − .339, *p* = .216, *n* = 15).

We additionally investigated the relation between vocal emotion recognition performance and symptom severity within the ASD group assessed by external rater (scores in communication and social interaction assessed by the ADOS, ADI-R, and SCQ). There was a significant correlation between the ADOS communication score and performance in the vocal emotion recognition test (*r* = − .672, *p* = .006, *n* = 15; all other *p*s > .078; Bonferroni corrected for the six scores at *p* = .008). There were no significant correlations between vocal pitch discrimination and symptom severity (all *p*s > .151).

### Influence of Emotional Intensity and Frequency Range on Vocal Emotion Recognition

ANOVAs for the factors group and emotional intensity/frequency range (see also Supplementary Material) revealed significant effects for the factors emotional intensity (*F*(3,28) = 53.529, *p* < .001) and frequency range (*F*(3,28) = 14.720, *p* < .001) on emotion recognition. There were no significant interactions between the factors group and emotional intensity (*F*(3,28) = 0.431, *p* = .733) or frequency range (*F*(3,28) = 1.622, *p* = .207).

## Discussion

Our study confirmed the hypothesis of a relation between vocal emotion processing abilities and pitch discrimination abilities in vocal sounds. There were three key findings. First, vocal emotion recognition abilities correlated with vocal pitch perception abilities in adults with typical development. There was no such significant correlation in adults with high-functioning ASD. However the correlation coefficients did not differ significantly between the two groups. Second, the ASD group performed worse than the comparison group in tests on vocal emotion recognition and on vocal pitch perception. There were no significant group differences in non-vocal pitch perception as assessed by the MBEA (Peretz et al. 2003, 2008) and no significant group differences in vocal timbre perception. Third, lower vocal emotion recognition abilities were associated with higher extents of autism spectrum related traits in people with typical development and showed a trend to an association with higher symptom severity in people with ASD.

Our findings are in line with the view that sensory processing differences in people with ASD might be critically contributing to difficulties in social functioning (Baum et al. 2015; Dakin and Frith 2005; Happe and Frith 2006; Pellicano and Burr 2012; Robertson and Baron-Cohen 2017). Differences in sensory processing, such as hypo- and hyper-sensitivity to sensory input, are part of the core symptoms of ASD (APA 2013). Previous studies mainly focused on hyper- and hypo-sensory processing which usually refers to an enhanced ability to perceive sensory stimuli or absent or less response to sensory input (for reviews see e.g. Pellicano 2013; Robertson and Baron-Cohen 2017). Other sensory processing difficulties might also be fundamentally contributing to difficulties in higher-level social cognition (for review see Baum et al. 2015). For example, previous behavioural and neuroimaging results on voice identity processing in people with ASD converge to the view, that difficulties in perceiving and processing acoustic voice features might at least partly explain difficulties in voice identity perception (Schelinski et al. 2016, 2017). Our current results now give first indications that the vocal emotion recognition difficulties of people with ASD might also be at least partly of perceptual nature. This is a novel view on the difficulties people with ASD have with vocal emotion recognition as previous studies rather focused on a dysfunction at a higher cognitive level (Globerson et al. 2015; Golan et al. 2007; Philip et al. 2010; Rutherford et al. 2002).

Our findings are in agreement with a previous study (Globerson et al. 2015) in that we found no significant group differences in *non-vocal* pitch perception abilities together with impaired vocal emotion recognition abilities in people with ASD. Critically, however, *vocal* pitch perception impairments were present together with vocal emotion processing difficulties in people with ASD. We speculate that people with typical development use vocal pitch information to perform vocal emotion recognition tests and that this is reflected in the correlation between vocal pitch processing and vocal emotion recognition abilities in the comparison group. That there was no such significant correlation in the ASD group might indicate that vocal pitch information is not available for recognition of vocal emotion to the same extent. As there was no significant difference in correlation strength for the correlation between vocal pitch perception and vocal emotion recognition abilities between the groups this assumption remains speculative and needs to be revalidated in bigger samples. However, our findings are important because they complement previous studies by providing evidence that difficulties in vocal emotion recognition in people with ASD might be due to impairments on the perceptual level and not only due to modality-independent social cognitive impairments as suggested previously (Globerson et al. 2015).

A previous study has indicated that people with ASD might use non-vocal pitch processing abilities as a compensatory mechanism to perform vocal emotion recognition (Globerson et al. 2015). Our finding that we did not find significant group differences in a standard test on non-vocal pitch perception abilities is in agreement with such a suggestion. We did, however, not find a correlation between non-vocal pitch and vocal emotion recognition. This difference between the Globerson et al. (2015) and our study might be explained by the use of different procedures to asses non-vocal pitch perception, i.e. an adaptive tracking procedure to determine individual thresholds in non-vocal pitch perception (Globerson et al. 2015) in contrast to recognition accuracy in a fixed set of stimuli in our study. Using an adaptive tracking procedure likely provides more sensitive results.

A prominent view on auditory processing in people with ASD suggests that difficulties in acoustic processing are more present for vocal stimuli (i.e. speech) as compared to non-vocal stimuli (i.e. non-speech) (e.g. see O’Connor 2012). In line with this assumption our ASD group had difficulties in vocal emotion and vocal pitch perception whereas the perception of non-vocal pitch (i.e. musical pitch assessed by the MBEA) was not significantly different between the groups. However, there are previous study results from adults with high-functioning ASD which contrast this assumption by showing: (i) Impairments in voice identity recognition that are dissociable from intact speech recognition abilities (Schelinski et al. 2016, 2014); (ii) Typical brain response to vocal sounds as compared to non-vocal sounds in voice-sensitive brain regions (Schelinski et al. 2016); and (iii) Intact vocal timbre perception (Bonnel et al. 2010) that is dissociable from difficulties in vocal pitch perception (see Table 4 and Schelinski et al. 2017). These results suggest that voice processing difficulties in people with high-functioning ASD do not cover all aspects of voice processing; they affect vocal pitch, vocal emotion and voice identity processing, but not to the same extent vocal timbre processing and vocal speech perception.

Previous studies showed that the expression (e.g. Nadig and Shaw 2012; for review see Fusaroli et al. 2017) and the perception of pitch can be altered in people with ASD (for review e.g. see O’Connor 2012). The ASD group showed significantly less accurate perception of vocal pitch than the comparison group whereas there were no significant group differences in non-vocal pitch perception (also see Schelinski et al. 2017). Our results on pitch perception are in line with previous evidence that non-vocal pitch perception (i.e. for pure and complex tones) is on the neurotypical level or even enhanced in people with ASD (e.g. Bonnel et al. 2003; Foxton et al. 2003; Globerson et al. 2015; Jones et al. 2009). With regard to vocal pitch perception previous results are less consistent (see e.g. Jarvinen-Pasley and Heaton 2007; Jiang et al. 2015). There are several factors that could explain the discrepancy between the findings, such as differences in the sample characteristics (e.g. differences in age or type of ASD diagnosis) and task design (e.g. differences in task difficulty and instruction or differences in the amount of pitch differences). Typical or even enhanced pitch processing in people with ASD has been related to a processing style which is characterised by enhanced or detailed perception of low-level perceptual information (enhanced perceptual functioning theory; Mottron et al. 2006) that can be associated with a weak ability to integrate elements into a coherent percept (weak central coherence theory; Happe and Frith 2006; for review see Haesen et al. 2011). While our results on vocal perception are difficult to explain by enhanced perception of low-level information, they are in line with the latter view and previous findings on voice identity perception (Schelinski et al. 2016, 2017) suggesting that difficulties in voice perception in people with high-functioning ASD might be related to difficulties in analysing and integrating complex acoustic voice features into a coherent voice percept.

Our results are in line with studies showing that in people with typical development vocal pitch information is essential for differentiating and recognising vocal emotion (e.g. Fairbanks and Pronovost 1938; Gold et al. 2012; Quam and Swingley 2012; Scherer et al. 1991). In the majority of these studies, the importance of vocal pitch in processing vocal emotion was shown by investigating how the perception of different emotions is influenced by different pitch characteristics of the vocal emotion stimulus material used in these studies. Here, we used an additional test on vocal pitch perception with independent stimulus material and provide first evidence that in people with typical development the ability to recognise vocal emotion is directly associated with the ability to perceive vocal pitch.

Previous studies showed that vocal emotion recognition difficulties are correlated with higher extents of autism spectrum traits as assessed by the AQ across people with typical development and people with ASD (Golan et al. 2006, 2007). However, it remained unclear whether such an association also holds when considering both groups separately. The present results indicated that vocal emotion recognition abilities were associated with AQ scores only within the comparison group. In line with previous study results (Rosenblau et al. 2017) within the ASD group, our results indicated a trend that vocal emotion recognition abilities were associated with symptom severity as assessed by the ADOS.

There are several possible confounds which mainly arise from task differences that we discuss in the following. For example, we assume that the differences in performance between vocal and non-vocal pitch perception in people with ASD is unlikely to be due to task differences as both tasks included complex sounds, i.e. vowels in the vocal pitch discrimination test and sounds from different instruments in the non-vocal pitch perception test. It is further unlikely that this dissociation in our study is due to differences in task difficulty as there were no group differences for the vocal timbre discrimination test which had exactly the same design as the vocal pitch discrimination test and only the task instruction differed. Critically, task differences, i.e. using an adaptive tracking procedure with pitch differences of less than one semitone, providing feedback after each response and conducting the test in the lab in the vocal pitch perception test might provide more sensitive results as compared to using a limited set of stimuli with pitch differences of at least one semitone in the non-vocal pitch perception test which was conducted online at home. We assume that this does not affect between group effects as both groups performed the tasks under the same conditions. However, the systematic investigation of vocal and non-pitch perception in people with ASD remains a subject to study. There are several other factors which might contribute to our results, such as verbal abilities, listener’s gender or the complexity of the presented emotions. For example there is evidence that verbal abilities are associated with vocal emotion recognition abilities, although findings are not consistent (for review see Lartseva et al. 2015). We assume that difficulties in vocal emotion recognition in our ASD sample cannot be explained by verbal abilities as groups were matched on verbal IQ and the same ASD group additionally showed intact speech recognition abilities and comparable speech sensitive brain responses as compared to the comparison group (Schelinski et al. 2016). Listener’s gender might be another critical variable which contributes to processing differences in emotion recognition (e.g. Rosenblau et al. 2017; Wacker et al. 2017). For example, a previous functional magnetic resonance imaging (fMRI) study showed differences in processing complex as compared to basic emotions in male and female participants (Rosenblau et al. 2017). We cannot infer on gender differences for the correlation between vocal emotion and vocal pitch discrimination based on the low number of females in our study. Further, we assume that the successful processing of complex emotions (e.g. pride, guilt) which requires a greater extent of socio-cognitive skills might at least partially underlie different mechanisms than we suggested for basic emotions (Alba-Ferrara et al. 2011; Rosenblau et al. 2017; Zinck and Newen 2008). The processing of vocal non-speech sounds (e.g. cry, laugh) which has been shown to be intact in people with ASD (Jones et al. 2011; Xavier et al. 2015) might also at least partially underlie different mechanisms. Additionally, one might assume that our study results are at least partly explainable by attention deficits within the ASD group. To control for possible attention differences between the ASD group and the comparison group, both groups were matched on attention using the d2 test of attention, i.e. there were no significant group differences in concentration performance as operationalised in this test. The d2 test relates to external visual stimuli. The ASD group might differ in the ability to attend stimuli using auditory stimuli. We find it however unlikely that a deficit in auditory attention can explain our results: We found comparable results between the ASD and the comparison group in tasks on working memory which required auditory attention and concentration, e.g. when recalling a series of numbers and letters which were read aloud by the experimenter (Wechsler 1997; Table 1). Additionally, there was a significant interaction in tasks with the same design and task demands (i.e. an interaction between vocal timbre and vocal pitch discrimination; Schelinski et al. 2017). Groups were also matched on performance IQ (Wechsler 1997), however, there was a larger variation of performance IQ scores within the ASD group. A pairwise matching with regard to performance IQ might additionally enhance comparability between the two groups.

We additionally tested whether the recognition accuracy in the vocal emotion recognition test was influenced by the level of emotional intensity or the frequency range of the stimulus material. Our results indicate that the overall worse performance in vocal emotion recognition in the ASD group was independent from the emotional intensity and frequency range of the stimuli used in the present study. This is in contrast to a previous study, in which vocal emotion recognition in people with ASD was mainly impaired for emotions that were difficult to recognise (low emotional intensity) and less impaired on emotion stimuli that were easy to recognise (high emotional intensity) (Globerson et al. 2015).

Behavioural data can provide evidence about possible underlying neuronal mechanisms. A previous study showed that the same sample of adults with high-functioning ASD as reported here, showed dysfunctional right posterior superior temporal sulcus and gyrus (STS/G) response to voice identity as compared to speech recognition (Schelinski et al. 2016; Supplementary Fig. 2). This region is in close proximity to posterior STS/G regions which preferably respond to vocal sounds including vocal speech and non-speech sounds (Belin et al. 2000), voice identity and vocal emotion processing in people with typical development (for meta-analyses see Blank et al. 2014; Frühholz and Grandjean 2013; Supplementary Fig. 2). Further, the posterior STS/G has been associated with sensitivity to acoustic aspects of the voice in vocal emotion (Frühholz et al. 2012) and voice identity perception (Andics et al. 2010; von Kriegstein et al. 2010; Warren et al. 2006). Thus, we speculate that difficulties in vocal emotion and voice identity recognition in people with high-functioning ASD might have a common origin in altered functioning of the posterior STS/G. However, the few studies that have so far investigated the brain representation of vocal emotion perception in people with ASD (Eigsti et al. 2012; Gebauer et al. 2014; Hesling et al. 2010; Rosenblau et al. 2017; Wang et al. 2007) do not provide clear evidence for altered functioning of the right posterior STS/G. Another candidate region for explaining difficulties in vocal pitch processing and potentially also vocal emotion recognition in people with ASD might be antero-lateral Heschl’s gyrus, because pitch processing is classically associated with this region (e.g. Kreitewolf et al. 2014; Patterson et al. 2002; Puschmann et al. 2010; for review see Griffiths and Hall 2012). However, it is currently unclear whether parts of antero-lateral Heschl’s specifically respond to vocal pitch. An explanation for the finding of vocal pitch processing deficits together with intact non-vocal pitch processing abilities in people with ASD at the level of antero-lateral Heschl’s is therefore highly speculative.

### Conclusion and Outlook

Difficulties in emotion recognition are socially restricting (Couture et al. 2006; Garcia-Villamisar et al. 2010) and associated with social difficulties in people with ASD (Boraston et al. 2007). Perceptual impairments might contribute significantly to difficulties in social cognition (Baum et al. 2015; Gold et al. 2012). In humans, the ability to adapt behaviour in accordance with the perceived vocal emotion in conspecifics develops early in infancy (Mumme et al. 1996; Vaish and Striano 2004; Walker-Andrews and Grolnick 1983; for review see Grossmann 2010). This suggests an important role of vocal emotion recognition in the development of social cognition. In people with ASD, difficulties in perceiving basic acoustic features, such as vocal pitch, likely contribute to the development of difficulties in higher-level social cognition, such as vocal emotion and voice identity perception. Together with other findings (Baum et al. 2015; Dakin and Frith 2005; Pellicano and Burr 2012; Schelinski et al. 2016, 2017), our results reveal that the investigation of lower-level sensory processing in people with ASD is important as such differences potentially underlie difficulties in higher-level social cognition. Furthermore the perception of lower-level sensory features might be a useful tool for the early diagnosis of ASD.

## Electronic supplementary material

Below is the link to the electronic supplementary material.

Supplementary material 1 (PDF 224 KB)
