# Supplementary material for: The Relation Between Vocal Pitch and Vocal Emotion Recognition Abilities in People with Autism Spectrum Disorder and Typical Development
Source: J Autism Dev Disord. 2018 Jul 18;49(1):68–82. doi: 10.1007/s10803-018-3681-z (PMC6331502; doi:10.1007/s10803-018-3681-z)
Supplement: Supplementary file 1 — Supplementary material 1 (PDF 224 KB) [file 10803_2018_3681_MOESM1_ESM.pdf]

The relation between vocal pitch and vocal emotion recognition abilities in people with autism spectrum disorder and typical development

Supplementary Material

Supplementary Methods

Behavioural and acoustic characterisation of the stimuli

Assessing the frequency range of the stimuli: We tested whether the recognition accuracy in the vocal emotion recognition test was influenced by the frequency range of the stimulus material. For this, we divided the stimuli in the vocal emotion recognition test into four equal quartiles based on their frequency range which we determined using Straight software (Kawahara & Irino, 2005) in a Matlab environment (version 7.7, The MathWorks, Inc., MA, USA). These quartiles represented stimuli with very low frequency range (up to 7 semitones), low frequency range (between 8 and 12 semitones), high frequency range (between 13 and 17 semitones), and very high frequency range (between 18 and 27 semitones).

Assessing the emotional intensity of the stimuli: We analysed whether the performance in the vocal emotion recognition test was influenced by the emotional intensity of the stimuli. To do that we calculated the recognition accuracy in the vocal emotion recognition test in an independent sample of adult participants with typical development (*n* = 21, 11 females, 10 males; mean age = 32.39; data reported in Roswandowitz et al., 2014). Based on the recognition rates of this independent comparison sample, we divided the stimuli of the vocal emotion recognition test into four quartiles with an equal number of stimuli. These quartiles represented stimuli with very low emotional intensity (recognition accuracy up to 72%), low emotional intensity (recognition accuracy between

73% and 84%), high emotional intensity (recognition accuracy between 85% and 92%), and very high emotional intensity (recognition accuracy between 93% and 100%).

Supplementary Figures

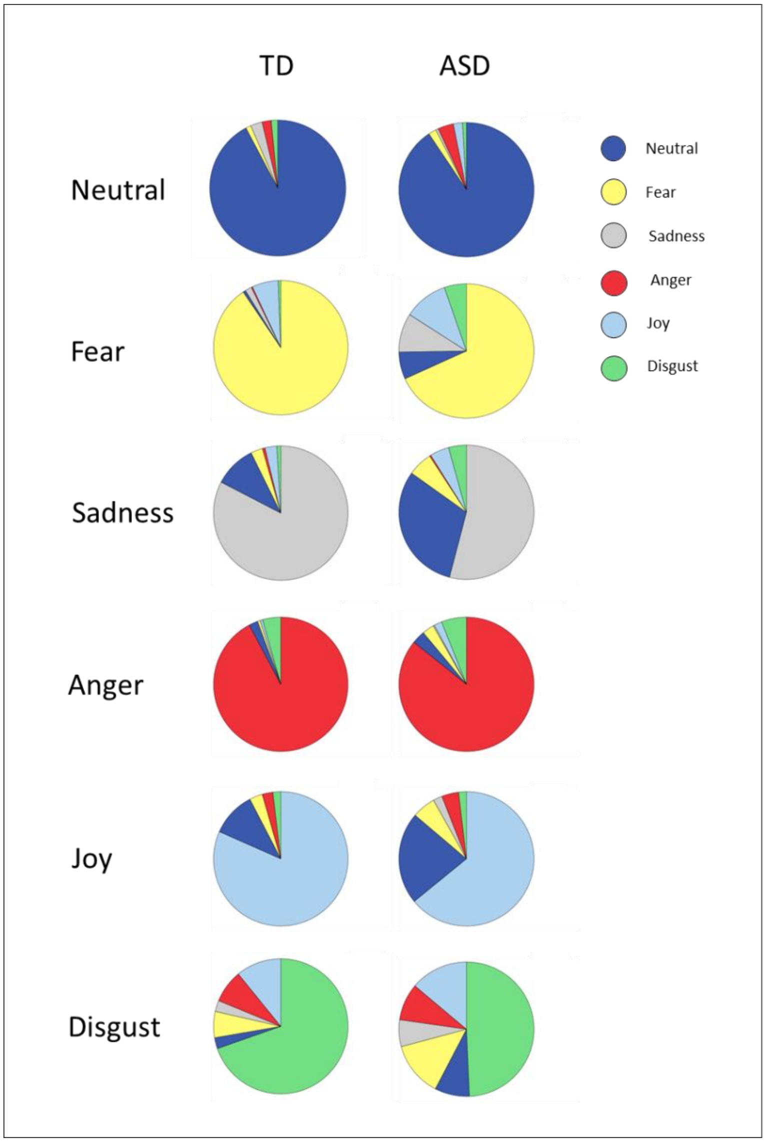

Supplementary Figure 1

Pie charts for the percentage of correct and incorrect (confusion) choices for each emotion for the comparison group with typical development (TD) and the ASD group.

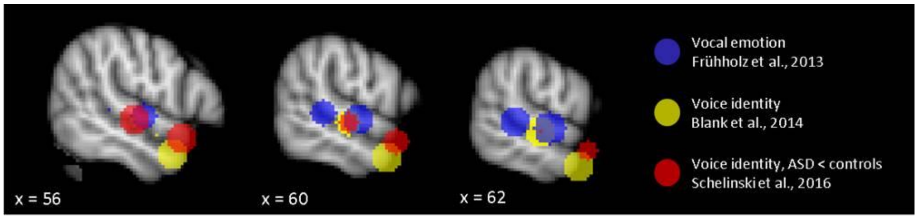

Supplementary Figure 2

Brain regions along the right temporal lobe that preferably respond to voice identity recognition (MNI-coordinates: x = 57, y = 6, z = -24; x = 65, y = -4, z = -12, and x = 66, y = -19, z = -2; coordinates taken from a meta-analysis (Blank et al., 2014) and vocal emotion in semantically neutral speech (MNI-coordinates: x = 62, y = -12, z = 0 and x = 66, y = -34, z = 4; coordinates taken from a meta-analysis (Frühholz & Grandjean, 2013). The high-functioning ASD group of the present paper showed reduced responses in similar brain regions when recognising speaker identity as compared to the comparison group (controls) (posterior: contrast voice identity > speech in controls vs. ASD group, MNI-coordinate: x = 51, y = -19, z = -2; anterior: correlation voice identity recognition with task performance in controls vs. ASD group, MNI-coordinate: x = 54, y = 11, z = -14 (Schelinski et al., 2016). For display purposes we created 10 mm spheres around the coordinates reported in the studies.

Supplementary Tables

Supplementary Table 1

Percentages of correct and incorrect (confusion) choices for each emotion for the comparison group and the ASD group.

**Comparison group**						
**Target**	Happiness	Sadness	Fear	Anger	Disgust	Neutral
**Response**						
Happiness	80,54	3,49	6,35	0,31	12,25	-
Sadness	0,31	81,64	1,89	-	2,86	2,50
Fear	3,44	2,58	89,85	0,31	7,91	0,94
Anger	1,90	0,63	0,31	92,48	7,55	1,88
Disgust	1,89	0,94	0,64	4,37	65,97	1,56
Neutral	11,91	10,72	0,95	2,51	3,45	93,13

**ASD group**						
**Target**	Happiness	Sadness	Fear	Anger	Disgust	Neutral
**Response**						
Happiness	64,10	4,46	10,64	1,88	14,18	2,19
Sadness	2,19	51,45	9,39	0,31	6,68	0,63
Fear	5,62	5,66	68,37	2,81	13,60	1,88
Anger	4,06	0,31	-	84,98	9,15	3,75
Disgust	1,88	4,18	5,33	5,95	50,18	0,94
Neutral	22,47	22,62	6,58	3,12	8,59	90,63

Supplementary References

Blank, H., Wieland, N., & von Kriegstein, K. (2014). Person recognition and the brain: merging evidence from patients and healthy individuals. *Neuroscience & Biobehavioral Reviews, 47C*, 717-734.

Frühholz, S., & Grandjean, D. (2013). Multiple subregions in superior temporal cortex are differentially sensitive to vocal expressions: a quantitative meta-analysis. *Neuroscience & Biobehavioral Reviews, 37*(1), 24-35.

Kawahara, H., & Irino, T. (2005). Underlying principles of a high-quality speech manipulation system STRAIGHT and its application to speech segregation. *Speech Separation by Humans and Machines*, 167-180.

Mugler, J. P. & Brookeman, J. R. (1990). Three-dimensional magnetization-prepared rapid gradient-echo imaging (3D MP RAGE). *Magnetic Resonance Medicine*, 15(1), 152-157.

Roswandowitz, C., Mathias, S. R., Hintz, F., Kreitewolf, J., Schelinski, S., & von Kriegstein, K. (2014). Two Cases of Selective Developmental Voice-Recognition Impairments. *Current Biology, 24*(19), 2348-2353.

Schelinski, S., Borowiak, K., & von Kriegstein, K. (2016). Temporal voice areas exist in autism spectrum disorder but are dysfunctional for voice identity recognition. *Social Cognitive and Affective Neuroscience, 11*(11), 1812-1822.
